# A Yeast Mutant Deleted of *GPH1* Bears Defects in Lipid Metabolism

**DOI:** 10.1371/journal.pone.0136957

**Published:** 2015-09-01

**Authors:** Martina Gsell, Ariane Fankl, Lisa Klug, Gerald Mascher, Claudia Schmidt, Claudia Hrastnik, Günther Zellnig, Günther Daum

**Affiliations:** 1 Institute of Biochemistry, Graz University of Technology, NaWi Graz, Petersgasse 12/2, 8010, Graz, Austria; 2 Institute of Plant Sciences, Karl Franzens University Graz, NaWi Graz, Austria; Simon Fraser University, CANADA

## Abstract

In a previous study we demonstrated up-regulation of the yeast *GPH1* gene under conditions of phosphatidylethanolamine (PE) depletion caused by deletion of the mitochondrial (M) phosphatidylserine decarboxylase 1 (*PSD1*) (Gsell et al., 2013, PLoS One. 8(10):e77380. doi: 10.1371/journal.pone.0077380). Gph1p has originally been identified as a glycogen phosphorylase catalyzing degradation of glycogen to glucose in the stationary growth phase of the yeast. Here we show that deletion of this gene also causes decreased levels of phosphatidylcholine (PC), triacylglycerols and steryl esters. Depletion of the two non-polar lipids in a Δ*gph1* strain leads to lack of lipid droplets, and decrease of the PC level results in instability of the plasma membrane. *In vivo* labeling experiments revealed that formation of PC via both pathways of biosynthesis, the cytidine diphosphate (CDP)-choline and the methylation route, is negatively affected by a Δ*gph1* mutation, although expression of genes involved is not down regulated. Altogether, Gph1p besides its function as a glycogen mobilizing enzyme appears to play a regulatory role in yeast lipid metabolism.

## Introduction

Cellular lipids fulfill three major functions. First, they provide a depot and source of energy especially in the form of triacylglycerols (TG) and steryl esters (SE) which are stored in lipid droplets [1]. Secondly, certain classes of lipids such as glycerophospholipids, sterols, sphingolipids and glycolipids are important components of biological membrane. Finally, lipids can act as cellular messengers [2]. Depending on the environmental and nutritional conditions one or the other function of lipids becomes paramount. Importantly, pathways of lipid storage/mobilization and membrane lipid biosynthesis are interlinked thus providing a means to switch within lipid metabolism to the required branches. As examples, fatty acids (FA) from TG and/or SE can either be used for energy production through β-oxidation or as building blocks for the synthesis of membrane lipids. Diacylglycerol (DG), the other degradation product of TG hydrolysis, can be utilized as a substrate for phospholipid (PL) synthesis and consequently a key intermediate in membrane lipid formation [2–6] but also as a second messenger.

The above mentioned scenario demonstrates that lipid metabolism is a highly complex network of reactions which are subject to sophisticated regulation. During the last few years our laboratory focused on the central role of phosphatidylethanolamine (PE), which is a key component in lipid metabolism. In the yeast *Saccharomyces cerevisiae*, the model organism which we use for our studies, PE is essential and one of the major phospholipids besides phosphatidylcholine (PC), phosphatidylinositol (PI) and phosphatidylserine (PS). The mitochondrial phosphatidylserine decarboxylase 1 (Psd1p) is the major producer of yeast PE catalyzing decarboxylation of PS to form PE [7,8]. The other three pathways of PE synthesis, namely decarboxylation of PS by Psd2p [9], the CDP-ethanolamine branch of the so-called Kennedy pathway [10], and synthesis of PE through acylation of lyso-PE catalyzed by the acyl-CoA-dependent acyltransferase Ale1p [11] are less efficient under standard growth conditions. Inactivation or deletion of the *PSD1* gene leads to a considerable decrease of PE in total cellular and especially in mitochondrial membranes and to a number of cellular defects [12,13].

To investigate effects of PE depletion caused by Δ*psd1* deletion on a genome wide basis we performed DNA microarray analysis of a Δ*psd1* mutant and compared its gene expression pattern with wild type [14]. This analysis revealed up-regulation of 54 genes in the Δ*psd1* mutant. One of the genes highlighted in this analysis was *GPH1*. Analysis of the growth phenotype and phospholipid composition suggested a possible involvement of *GPH1* in lipid metabolism. Gph1p was originally identified as glycogen phosphorylase 1 catalyzing the release of glucose 1-phosphate from glycogen in the late stationary growth phase of the yeast to maintain the required energy for cell activity and growth during periods of nutrient starvation [15–19]. The activity of glycogen phosphorylase is regulated by cyclic AMP-mediated phosphorylation of the enzyme. *GPH1* is not essential in yeast, but Δ*gph1* mutants lacking the phosphorylase activity exhibit increased levels of intracellular glycogen [15].

In previous studies it has been shown that Gph1p is localized on so-called glycogen particles [15,20] whose size and amount vary during the growth phases of the yeast cell [14]. Glycogen, which is a storage form of carbon and energy, consists of branched glucose polymers and is synthesized by many different organisms [20]. Degradation of glycogen occurs when energy is required for cell activity and growth during stationary phase. In the yeast, expression of *GPH1* is induced at the end of the logarithmic growth phase. Almost simultaneously intracellular glycogen starts to accumulate as long as carbon sources are present. This finding suggested an important role of Gph1p in glycogen utilization as a reserve energy source during periods of nutrient starvation. Gph1p is inhibited by glucose 6-phosphate, and its activity is regulated by reversible phosphorylation [15]. The Δ*gph1* deletion mutant accumulates large amounts of glycogen during the stationary phase and shows rapid chronological aging and low stress tolerance [16].

In the present study, the influence of high glycogen content in the Δ*gph1* mutant was compared to wild type and to *Δglc3*, a strain with inhibited glycogen synthesis [21]. Moreover, the influence of *GPH1* overexpression on phospholipid pattern and glycogen content was analyzed and compared to the other mutant strains. Analysis of Δ*gph1*, *Δglc3* and wild type overexpressing *GPH1* [pYES_gph1] revealed changes in lipid metabolism of the mutants besides the above mentioned effects. In particular, a Δ*gph1* yeast mutant exhibited decreased PC levels in total cell homogenates (H) and especially in the plasma membrane as well as changes in the metabolism of non-polar lipids. Thus, it appears that Gph1p is involved in several branches of lipid metabolism in addition to its role as global player in carbohydrate metabolism. The multiple functions of Gph1p with emphasis on regulatory aspects in lipid metabolism are discussed in this paper.

## Materials and Methods

### Strains and media

Yeast strains used in this study are listed in Table 1. Cells were grown in liquid YPD media (1% yeast extract, 2% peptone and 2% glucose) under aerobic conditions with shaking at 30°C. Growth tests on solid media were performed on 1% yeast extract, 2% peptone, and 2% agar supplemented with 2% glucose, 2.66% lactate (adjusted to pH 5.5 with KOH), 2% glycerol or 8 mM sorbitol, respectively. For SDS resistance assays on solid media, 0.05% SDS was added to the media immediately prior to pouring plates. MMLac (minimal medium with lactate) culture plates contained 0.67% yeast nitrogen base without amino acids, 0.073% amino acid mix, 2.66% lactate, adjusted to pH 5.5 with KOH, and 2% agar.

**Table 1:** Yeast strains used in this study.

Strain	Genotype	Source / reference
BY4741	Mata *his3*Δ1 *leu2*Δ0 *met15*Δ0 *ura3*Δ0	Euroscarf (Frankfurt, Germany)
Δ*gph1*	Mata *his3*Δ1 *leu2*Δ0 *met15*Δ0 *ura3*Δ0 *gph*1Δ::KanMX4	Euroscarf (Frankfurt, Germany)
Δ*psd1*	Mata *his3*Δ1 *leu2*Δ0 *met15*Δ0 *ura3*Δ0 *psd1*Δ::KanMX4	Euroscarf (Frankfurt, Germany)
Δ*gph1*Δ*psd1*	Mata *his3*Δ1 *leu2*Δ0 *met15*Δ0 *ura3*Δ0 *gph1*Δ::KanMX4 *psd1*Δ::His3MX6	This study
*Δsga1*	Mata *his3*Δ1 *leu2*Δ0 *met15*Δ0 *ura3*Δ0 *sga*1Δ::KanMX4	Euroscarf (Frankfurt, Germany)
*Δgbd1*	Mata *his3*Δ1 *leu2*Δ0 *met15*Δ0 *ura3*Δ0 *gbd*1Δ::KanMX4	Euroscarf (Frankfurt, Germany)
*Δglc3*	Mata; *his3*Δ1 *leu2*Δ0 *met15*Δ0 *ura3*Δ0; YEL011w::kanMX4	Euroscarf (Frankfurt, Germany)
*BY4741 [pYES2]*	*Mata his3Δ1 leu2Δ0 met15Δ0 ura3Δ0 + pYES2*	This study
*BY4741 [pYES2_gph1]*	*Mata his3Δ1 leu2Δ0 met15Δ0 ura3Δ0 + pYES2-gph1*	This study

Yeast strains transformed with expression plasmids were grown on minimal medium minus uracil containing 2% galactose (Roth), 0.67% yeast nitrogen base without amino acids (U.S. Biological) and 0.063% amino acid mix without uracil (Roth, Fluka).

For growth phenotype analysis cell suspensions of overnight cultures grown on YPD were spotted at dilutions 1, 1/10, 1/100, 1/1000 and 1/10000 on YPD, YPLac, MMLac, YPGlycerol, YPSorbitol, and YPD with 0.05% SDS, respectively. Incubations were carried out at 30°C.

### Plasmid and strain constructions

Inactivation of the *PSD1* gene in a Δ*gph1* deletion mutant was performed by transformation of the Δ*psd1*::His3MX6 disruption cassette as described by Longtine et al. [22]. The His3MX6 disruption cassette was amplified from the pFA6a-His3MX6 plasmid using the primers PSD1-koF 5’- gccagttaagaacgccttggcgcaagggaggacgctcctccggatccccgggttaattaa-3’ and PSD1-koR 5’- caggtatgtggttccaagtgtttgtcgc -3’.

The disruption cassette was amplified by PCR under standard conditions using the proof reading *Ex Taq*-DNA polymerase (Takara). The cassette was introduced into the respective strain by lithium acetate transformation [23]. Correct insertion of the cassette was tested by growing strains on selective media. Identity of strains was confirmed by marker-dependent growth, colony PCR and sequencing.

Yeast strains expressing *GPH1* were generated by amplifying *GPH1* using gene-specific primers from genomic DNA in a standard PCR mixture containing Ex Taq-DNA polymerase (Takara). The PCR product was purified and inserted via BamHI and XhoI (Fermentas) restriction sites into the pYES2 vector (Invitrogen), forming pYES2-GPH1.

### RNA isolation and Real-Time PCR

For RNA isolation, cells were grown to the mid-logarithmic growth phase on YPD at 30°C. Total RNA was isolated using an RNeasy kit from Qiagen following the manufacturer’s instructions. After DNaseI digestion, Real-Time PCR was performed using the SuperScript III Platinum SYBR Green One-Step qRT-PCR Kit (Invitrogen) as described by the manufacturer. Amplification was measured by using an ABI 7500 instrument (Applied Biosystems) in sealed MicroAmp Optical 96-Well Reaction Plates. Relative quantities of RNA were determined using the ΔΔCt method described by Livak and Schmittgen [24]. Differences in mRNA expression were calculated after normalization with *ACT1* relative to wild type control. Primers used for RT-PCR are listed in Table 2.

**Table 2:** Primers used for RT-PCR.

Primer	Sequence (5’→ 3’)
RT Act1-fwd	CCAGCCTTCTACGTTTCCATCCAAG
RT Act1-rev	GACGTGAGTAACACCATCACCGGA
RT Tgl3-fwd	GCCAACAATCCGAGCATAACGGAG
RT Tgl3-rev	TGGTGCCAAGTATGGTCTCGCCA
RT-Tgl4-fwd	TGCCCGACATGTGTATGCTTTTTAGAAT
RT-Tgl4-rev	CTTGGGCCACGTAGCTTTTGCAC
RT-Tgl5-fwd	CCGGGAGTTGACTTGGAAGAATCC
RT-Tgl5-rev	GGAGAAGGCAATGGCTGAAGAGGA
RT-Psd1fwd	GCCTCATGATACGGAACTTTTCTTTGC
RT-Psd1rev	CTGGGAAATGGCGGCGAAC
RT-Psd2fwd	GGCGCCACAAGATTATCACCGGTT
RT-Psd2rev	CGGCCATTGGATTTACAGTATAATA
RT-Cho2fwd	ATCGTGAAAAAACAAGAGTTGGATCAGGT
RT-Cho2rev	CCTTGAACGTACTTTTCAGTCGCCTTT
RT-Opi3fwd	ACCAAGCTGGGTGTGGCTCTCTTT
RT-Opi3rev	TCTCTCATCCATCAGGATGCCGAAATA

### Calcofluor White sensitivity test

Sensitivity of yeast cells against Calcofluor White was tested as described by Takahashi et al. [25]. Cells were grown over night in YPD medium at 30°C with shaking. One OD_600_ unit was harvested, washed once with sterile water and diluted with 1 mL sterile water. Cell suspensions were spotted at dilutions of 1, 1/10, 1/100, 1/1000, 1/10000 onto YPD agar plates containing 20 μg/ml or 30 μg/ml Calcofluor White (CFW) and incubated at 30°C for 3 days.

### Isolation of plasma membrane and mitochondria

Yeast cells were grown aerobically in YPD to the early stationary growth phase at 30°C. Then, cells were disrupted with glass beads using a Merckenschlager homogenizer under CO_2_-cooling. Cell extracts were cleared of glass beads, unbroken cells and cell debris by centrifugation at 2500 x g for 5 min. The supernatant fraction represented the homogenate. Crude plasma membrane was isolated essentially as described by Serrano [26] and further purified as reported by van den Hazel et al. [27] and Pichler et al. [28].

To isolate mitochondria, spheroplasts were prepared and homogenized in breaking buffer consisting of 0.6 M mannitol, 10 mM Tris, pH 7.4, and 1 mM phenylmethanesulfonylfluoride (PMSF) by using a Dounce homogenizer as described previously [29]. Unbroken cells and debris were removed by centrifugation at 3,000 x g for 5 min. The resulting supernatant was used to isolate mitochondria by published procedures [30].

Relative enrichment of markers and cross-contamination of subcellular fractions were assessed as described by Zinser and Daum [31]. Protein was quantified by the method of Lowry et al. [32] using bovine serum albumin (BSA) as standard. SDS-PAGE was carried out as published by Laemmli [33]. Western blot analysis of proteins from subcellular fractions was performed as described by Haid and Suissa [34]. Immunoreactive bands were visualized by ELISA using a peroxide-linked secondary antibody (Sigma-Aldrich, St. Louis, MO) following the manufacturer´s instructions.

### Lipid analysis

Lipids from yeast cells were extracted as described by Folch et al. [35]. For phospholipid analysis 3 mg protein from total cell homogenate or 2 mg protein from mitochondria and plasma membrane fractions, respectively, were extracted using 3 ml chloroform/methanol (2:1; v/v). Individual phospholipids were separated by two-dimensional thin-layer chromatography (TLC) on silica gel 60 plates (Merck, Darmstadt, Germany) using chloroform/methanol/25% NH_3_ (50:25:6; per vol.) as first, and chloroform/acetone/methanol/acetic acid/water (50:20:10:10:5; per vol.) as second developing solvents. Phospholipids were stained with iodine vapor, scraped off the plate and quantified by the method of Broekhuyse [36].

For fatty acid analysis, cells were harvested during the logarithmic growth phase. After cell disruption using glass beads, the homogenate containing 1 mg protein was used for fatty acid analysis. Lipids were extracted as described above and fatty acids were converted to fatty acid methyl esters by methanolysis using 2.5% sulfuric acid in methanol and heating at 80°C for 90 min. Fatty acid methyl esters were extracted in a mixture of light petroleum and water (3/1; v/v) and analyzed by gas liquid chromatography (Hewlett-Packard 6890 Gas-chromatograph) using a HP-INNOWax capillary column (15 m × 0.25 mm i.d. × 0.50 μm film thickness) with helium as carrier gas. Fatty acids were identified by comparison to the fatty acid methyl ester standard mix GLC-68B (NuCheck, Inc., Elysian, MN, USA) and hexacosanoic acid methyl ester standard (Sigma Aldrich, Vienna).

For quantification of non-polar lipids, lipid extracts were applied to Silica Gel 60 plates, and chromatograms were developed in an ascending manner by a two-step developing system [37]. First, chromatograms were developed using light petroleum/diethyl ether/acetic acid (70:30:2; v/v) and then light petroleum:diethyl ether (49:1; v/v) as solvents. To visualize separated bands, TLC plates were dipped into a charring solution consisting of 0.63 g MnCl_2_ x 4 H_2_O, 60 ml water, 60 ml methanol and 4 ml concentrated sulfuric acid, briefly dried and heated at 100°C for 20 min. Then, lipids were quantified by densitometric scanning at 400–650 nm with triolein, cholesteryl esters and ergosterol (ERG) as standards using a Shimadzu dual-wave length chromatoscanner CS-930. For DG analysis chromatograms were developed in chloroform/acetone/acetic acid (45:4:0.5; per vol.) with diolein as standard.

### Analytical procedures

For the analysis of glycerol 3-phosphate (G3P), 10 OD_600_ units of yeast cells were harvested and lysed using the cellLytic Y-Yeast cell Lysis reagent (Sigma–Aldrich). Cells were incubated with 100 μl lysis buffer for 15–30 min at room temperature and centrifuged for 10 min to remove cell debris. For wild type analysis 10–20 μl, and for the analysis of *Δgph1* 2.5–5 μl of the supernatant were used for the glycerol 3-phosphate assay using the Glycerol 3-phosphate Colorimetric Assay Kit (Sigma-Aldrich).

For qualitative analysis of glycogen, cells were grown to the stationary phase and spotted onto YPD plates. Plates were grown for 48 h at 30°C prior to detection of glycogen by exposing plates to iodine vapor [38]. The intensity of the brown color was used as indicator for the cellular glycogen content.

Analysis of glycogen was performed following the instructions of the manual of the Glycogen Assay Kit (Sigma–Aldrich). For this purpose, 2 OD_600_ units of yeast cells were harvested and lysed using the cellLytic Y-Yeast cell Lysis reagent (Sigma–Aldrich). Cells were incubated with 20 μl lysis reagent for 15–30 min at room temperature and centrifuged for 10 min to remove cell debris. For the wild type strain 5 μl, and for the *Δgph1* strain 2.5 μl of homogenate were used for glycogen analysis.

### Metabolic labeling of phospholipids and non-polar lipids

Labeling of aminoglycerophospholipids *in vivo* was determined by following the incorporation of L-[^3^H]serine or [methyl-^14^C]choline chloride, respectively, into PS, PE and PC as described previously [13,39]. An equivalent of 10 OD_600_ units from an overnight culture (~1 ml, corresponding to 1.45 x 10^8^ cells) was harvested in a Pyrex tube, washed once, suspended in 500 μl YPD and incubated for 30 min at 30°C. Cells were labeled with 10 μCi [³H]serine (21.99 Ci·mmol^-1^, Perkin-Elmer, Boston, MA) or 10 μCi [methyl-^14^C]choline chloride (54 mCi·mmol^-1^, Perkin Elmer Boston, MA), respectively, for 1 h at 30°C. Samples were put on ice, harvested by centrifugation and shock frozen with liquid nitrogen. Chloroform/methanol (2:1; v/v) and glass beads were added to the cell pellets and samples were vigorously shaken on an IKA Vibrax VXR for 1 h. Then, lipids were extracted by the method of Folch et al. [35]. Individual phospholipids were separated by TLC on Silica gel 60 plates (Merck, Darmstadt, Germany) with chloroform/methanol/25% NH_3_ (50:25:6, per vol.) as developing solvent. Spots on TLC plates were stained with iodine vapor, scraped off and suspended in 8 ml scintillation cocktail (Packard Bio-Science, Groningen, the Netherlands) containing 5% water. Radioactivity was determined by liquid scintillation counting using a Packard TriCarb Liquid Scintillation Analyzer.

To estimate the incorporation of [1-^14^C]acetic acid *in vivo* into total phospholipids and non-polar lipids an equivalent of 10 OD_600_ from an overnight culture was harvested, washed and suspended in 500 μl YPD. After an incubation of 30 min at 30°C, cells were labeled with 0.5 μCi [1-^14^C]acetic acid (55.3 mCi mmol^-1^, Perkin Elmer, Boston, MA) for 0, 10, 20, 30, 60 and 120 min, respectively. Lipids were extracted as described above, and individual lipids were separated by TLC with light petroleum/diethyl ether/acetic acid (70:30:2; per vol.) and then light petroleum/diethyl ether (49:1; per vol.) as solvents. Radioactivity was determined as described above.

### In vivo mobilization of non-polar lipids

To measure the mobilization of non-polar lipids, cells were pre-grown for 24 h in minimal medium containing 0.67% yeast nitrogen base without amino acids, 0.073% amino acid mix and 2% glucose as the carbon source. Then, fresh minimal medium was inoculated with the pre-grown culture to an OD_600_ of 3, and cerulenin (final concentration 10 μg/ml) was added from an ethanolic stock solution. Control incubations contained the equivalent volume of ethanol only. At time points indicated, aliquots of the culture were withdrawn, and an equivalent of 10 OD_600_ were harvested by centrifugation on a table-top centrifuge. The pellet was washed and shock frozen with liquid nitrogen. Lipids were extracted and analyzed as described above [40].

### Microscopy of yeast cells

For electron microscopic inspection, cells were harvested in the early stationary growth phase by centrifugation and washed three times with double-distilled water. Subsequently, cells were fixed for 5 min in a 1% aqueous solution of KMnO_4_ at room temperature, washed with distilled water, and fixed in a 1% aqueous solution of KMnO_4_ for 20 min again. Fixed cells were washed three times in distilled water and incubated in 0.5% aqueous uranylacetate overnight at 4°C. Samples were then dehydrated for 20 min in a graded series of 50%, 70%, 90% and 100% ethanol, each. Pure ethanol was then changed to propylene oxide, and specimen were gradually infiltrated with increasing concentrations (30%, 50%, 70% and 100%) of Agar 100 epoxy resin mixed with propylene oxide for a minimum of 3 h per step. Samples were finally embedded in pure, fresh Agar 100 epoxy resin and polymerized at 60°C for 48 h. Ultrathin sections of 80 nm were stained with lead citrate and viewed with a Philips CM 10 transmission electron microscope.

For fluorescence microscopy of lipid droplets Nile Red staining was performed as described by Greenspan et al. [41]. Yeast strains were grown to the early stationary growth phase and stained with Nile Red dissolved in ethanol. Microscopic pictures were visualized using a fluorescence microscope (Axiovert 35, Carl Zeiss, Jena, Germany) with the filter set 14 (Zeiss). Nile Red fluorescence of lipid droplets was detected at an emission wavelength of 590 nm with a 100-fold oil immersion objective.

## Results

### Growth phenotype analysis

In a previous study from our laboratory [14] a genome wide approach revealed genetic and functional interaction of *GPH1* and *PSD1* from the yeast *Saccharomyces cerevisiae*. To obtain insight into the possible involvement of the *GPH1* gene product in lipid metabolism and especially in PE synthesis we performed a number of tests including growth behavior and lipid profiling of a Δ*gph1* mutant strain. In brief, we showed that Δ*gph1* and Δ*psd1* mutants grew like wild type on glucose-containing media. On non-fermentable carbon sources (YPLac and YPGlycerol), however, growth of Δ*psd1* was markedly reduced, whereas the Δ*gph1* mutant grew like wild type. In the present study, growth tests were extended to the Δ*gph1*Δ*psd1* double mutant on both full media, but results largely reflected the effects of the Δ*psd1* deletion (data not shown). Growth on sorbitol containing plates revealed that Δ*gph1* as well as Δ*psd1* mutants became slightly instable [14]. Most interestingly, the Δ*gph1* deletion mutant was highly sensitive to SDS. Such an effect was not observed with the Δ*psd1* strain. This result suggested that in Δ*gph1* most likely the cell surface, the plasma membrane and/or the cell wall were compromised. In line with this view we showed that the phospholipid composition of the plasma membrane was compromised by the Δ*gph1* mutation, especially by decreasing dramatically the PC to PE ratio in this compartment [14].

**Fig 1:**
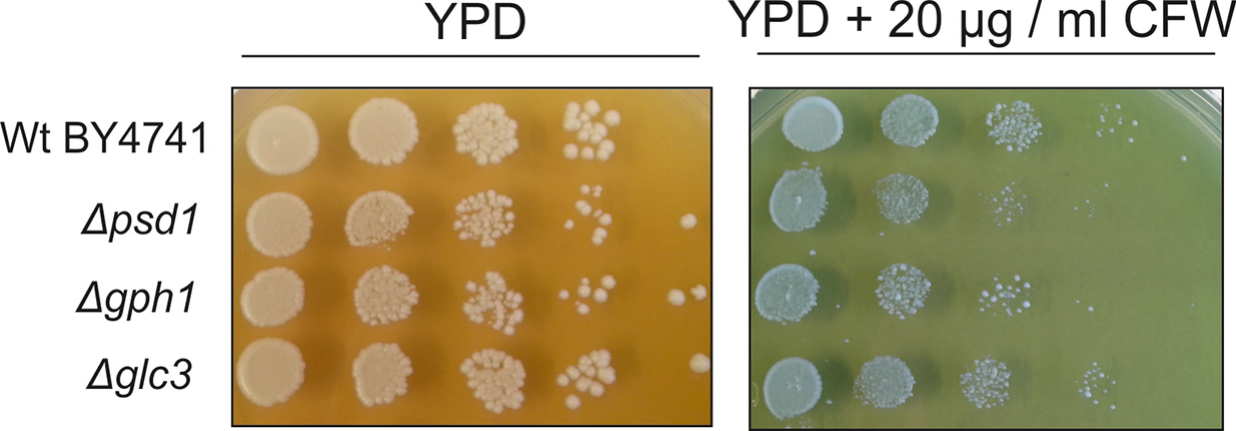
Calcofluor White sensitivity of *Δgph1*, *Δpsd1* and *Δglc3* deletion mutants. Wild type BY4741 and mutant strains as indicated were grown on YPD plates and on YPD plates containing 20 μg/ml Calcofluor White (CFW).

To further test the sensitivity of the plasma membrane from a Δ*gph1* mutant strain we performed tests with Calcofluor White (CFW), which serves as a sensor for abnormalities of the plasma membrane [25]. Interestingly, CFW had a slight effect on the growth behavior of *Δgph1* and *Δpsd1* (Fig 1). As a control, we also tested the effect of CFW on a *Δglc3* strain which bears a mutation in glycogen synthesis [21]. However, growth of *Δglc3* was also unaffected by CFW. Even higher concentrations of CFW, e.g., 30 μg/ml did not influence the growth behavior of all strains tested (data not shown).

### Phospholipid analysis of total cell homogenate and subcellular fractions

**Fig 2:**
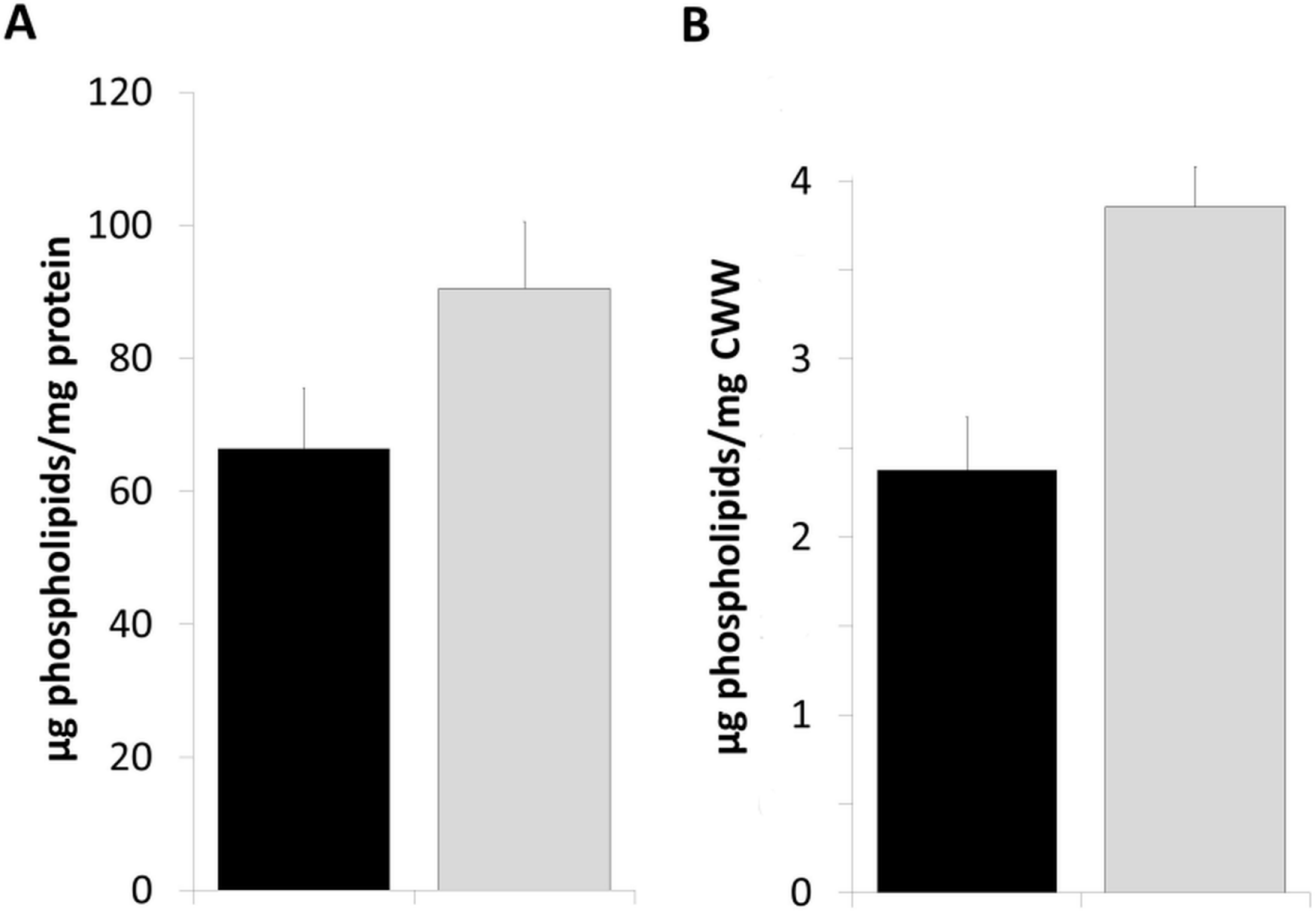
Total amount of phospholipids in cell free homogenate from wild type and the Δ*gph1* deletion mutant. Cells were grown on YPD to the early stationary growth phase at 30°C and disrupted with the aid of glass beads. Lipids were extracted with chloroform/methanol (2:1; v/v). Black bar, wild type BY4741; grey bar, Δ*gph1* mutant. (A) Total amounts of phospholipids were related to the amounts of protein. (B) Total amounts of phospholipids were related to cell wet weight. Inorganic phosphate was used as standard.

In our previous study [14] we showed that a Δ*gph1* mutation affected phospholipid profiles of total cell homogenate, mitochondria and especially the plasma membrane from cells grown on complex YPD media. The most striking effect was depletion of PC in the latter compartment. To rule out the possibility that total phospholipid production was compromised in a *Δgph1* we quantified the total amount of phospholipids in wild type and in the mutant strain (Fig 2). As can be seen the *Δgph1* mutation rather led to an increased phospholipid level arguing against a negative influence of the mutation on the formation of total cellular membranes.

In the present study we extended the lipid profiling to a strain overexpressing *GPH1* (Table 3; WT [pYES_gph1]). In contrast to Δ*gph1*, overexpression of the gene led to a slight increase of PC in the total cell extract and in mitochondria. Surprisingly, the plasma membrane of WT [pYES_gph1] was rather depleted of PC. As another control, we analyzed lipids from a *Δglc3* mutant (see Table 3). The *GLC3* gene product (1,4-glucan-6-(1,4-glucano)-transferase) is involved in glycogen metabolism catalyzing the last step in the biosynthetic pathway. As can be seen from Table 3, a *Δglc3* deletion led to slight accumulation of PC in the total cell extract and a marked increase of PC in the plasma membrane, whereas a marked decrease of PC in the mitochondria was observed. Thus, in comparison with *Δgph1* [14], it appears that there is no strict correlation between glycogen metabolism and PC biosynthesis. This view was confirmed by analysis of *sga1Δ* and *gdb1Δ* deletion strains. The respective gene products of *SGA1* (glucoamylase) and *GDB1* (glucotransferase) are involved in glycogen biosynthesis similar to Gph1p. However, both deletions had only minor and non-significant effects on PC formation (data not shown).

**Table 3:** Phospholipid composition of cell-free homogenate, plasma membrane and mitochondria from cells grown on YPD or minimal medium (*). CF, cellular fraction; LPL, lysophospholipids; PI, phosphatidylinositol; PS, phosphatidylserine; PE, phosphatidylethanolamine; PC, phosphatidylcholine; CL, cardiolipin; DMPE, dimethylphosphatidylethanolamine; PA, phosphatidic acid. WT [pYES2], wild type bearing plasmid pYES2; WT [pYES_gph1], overexpression of *GPH1* on plasmid pYES in wild type background. Mean values of at least three measurements and standard deviations are shown.

		Phospholipids (mol%)								
CF	Strain	LPL	PI	PS	PC	PE	CL	DMPE	PA	Others
Homogenate	WT [pYES2]*	1.9 ± 1.1	7.5 ± 1.0	8.0 ± 0.8	47.5 ± 3.9	24.5 ± 5.7	3.0 ± 0.9	1.1 ± 0.9	3.6 ± 1.4	3.1 ± 0.9
	WT [pYES_gph1]*	1.4 ± 0.9	7.5 ± 1.1	6.7 ± 0.9	53.2 ± 5.9	21.8 ± 1.4	3.2 ± 0.3	1.8 ± 0.3	2.8 ± 0.8	1.5 ± 0.5
	WT	1.5 ± 0.2	9.9 ± 3.5	8.8 ±.0.5	45.1 ± 1.8	26.6 ± 2.5	3.4 ± 0.3	4.4 ± 0.7	0.7 ± 0.4	0.0 ± 0.0
	*Δglc3*	0.1 ± 0.0	7.8 ± 3.2	5.4 ± 1.5	47.3 ± 5.1	30.4 ± 5.5	3.3 ± 1.2	4.3 ± 1.4	1.4 ± 0.8	0.0 ± 0.0
Mitochondria	WT [pYES2]*	3.5 ± 0.8	6.6 ± 1.3	3.9 ± 0.4	37.1 ± 8.6	39.6 ± 7.3	5.5 ± 1.9	0.3 ± 0.0	2.8 ± 2.2	0.8 ± 0.2
	WT [pYES_gph1]*	3.5 ± 0.7	6.1 ± 0.4	2.5 ± 0.7	41.5 ± 9.5	35.6 ± 6.2	7.3 ± 2.0	1.2 ± 1.0	1.5 ± 0.9	1.0 ± 0.5
	WT	1.9 ± 1.1	8.1 ± 1.7	4.1 ± 0.6	40.7 ± 2.4	30.4 ± 1.4	5.0 ± 3.6	6.6 ± 3.5	2.4 ± 0.4	0.7 ± 0.6
	*Δglc3*	0.6 ± 0.1	6.5 ± 2.7	2.9 ± 0.3	27.5 ± 5.0	41.0 ± 4.3	9.1 ± 4.3	4.3 ± 0.9	8.1 ± 1.2	0.0 ± 0.0
Plasma membrane	WT [pYES2]*	2.3 ± 0.7	4.7 ± 0.3	15.9 ± 0.5	35.4 ± 5.7	33.3 ± 5.1	0.0 ± 0.0	0.6 ± 0.4	4.1 ± 0.9	3.6 ± 0.2
	WT [pYES_gph1]*	8.2 ± 1.7	9.3 ± 5.2	24.9 ± 7.8	19.5 ± 6.5	31.0 ± 9.8	3.0 ± 0.5	0.0 ± 0.0	4.3 ± 1.0	0.0 ± 0.0
	WT	2.4 ± 1.0	12.4 ± 2.1	26.2 ± 2.8	18.2 ± 1.4	32.1 ± 2.1	0.7 ± 0.1	2.2 ± 0.4	5.8 ± 0.6	0.0 ± 0.0
	*Δglc3*	0.8 ± 0.2	7.7 ± 5.7	13.1 ± 2.3	42.3 ± 8.9	28.3 ± 6.5	0.5 ± 0.3	5.9 ± 2.9	1.4 ± 0.4	0.0 ± 0.0

### Synthesis of PC is down-regulated in the *Δgph1* deletion mutant in vivo

**Fig 3:**
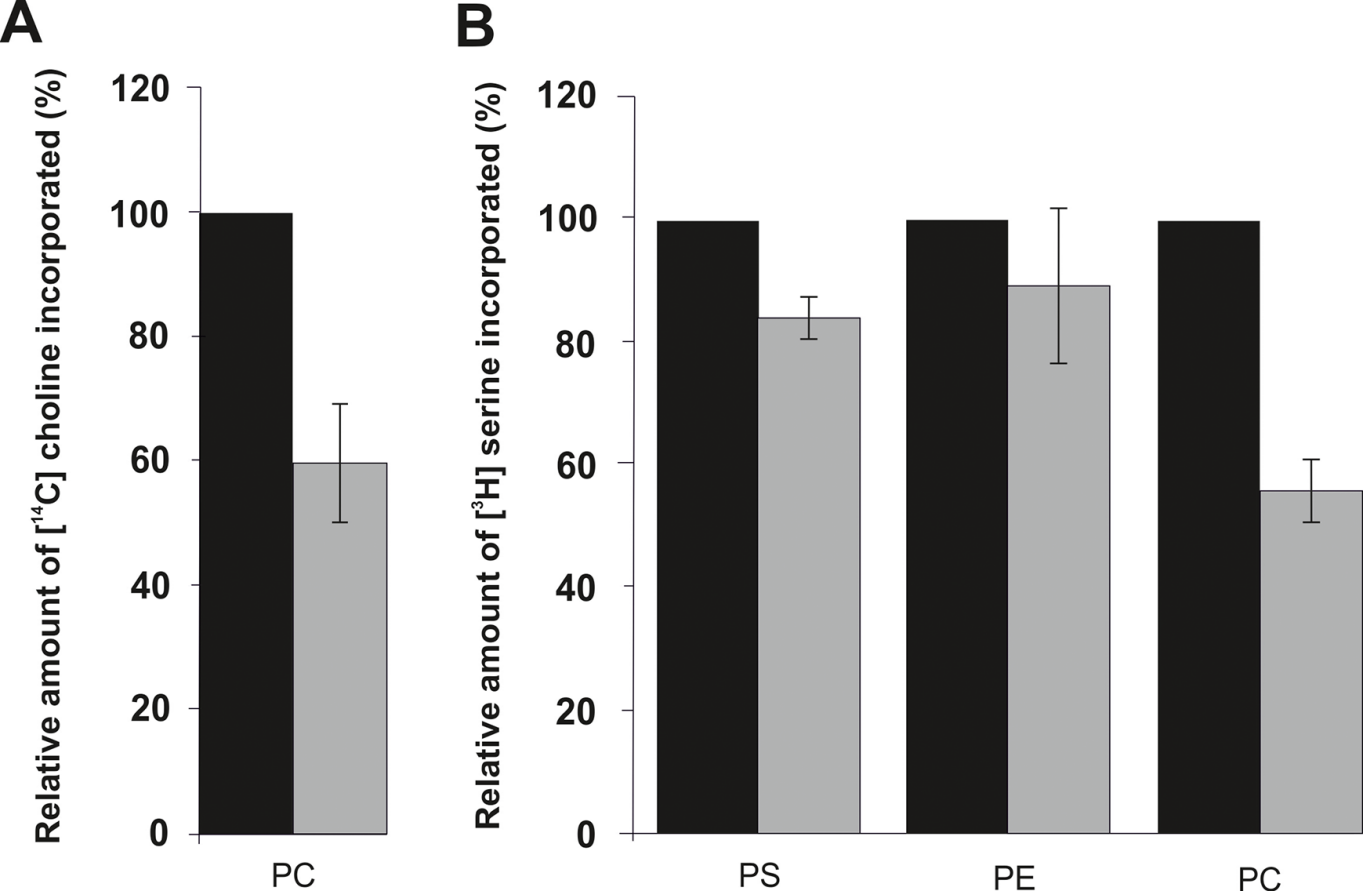
Synthesis of phosphatidylcholine is down regulated in the Δ*gph1* deletion mutant *in vivo*. Black bar, wild type BY4741; grey bar, Δ*gph1* deletion mutant; (A) The so-called Kennedy pathway (CDP-choline pathways) was analyzed by incorporation of [methyl-^14^C]choline chloride into PC. (B) To analyze the methylation pathway of PC, the incorporation of L-[^3^H]serine into PS, PE and PC was measured. Wild type BY4741 was set at 100%.

As described above, the PC level in the Δ*gph1* deletion mutant was markedly decreased compared to wild type. As there are two pathways of PC synthesis in *Saccharomyces cerevisiae* we wished to estimate which one was affected by the Δ*gph1* deletion. PC can be synthesized (i) via the CDP-choline branch of the Kennedy pathway which utilizes choline as a substrate [10]; or (ii) through a three step methylation of PE catalyzed by Cho2p and Opi3p [42–44]. To analyze the CDP-choline pathway we labeled cells with [methyl-^14^C]choline chloride and measured incorporation of the label into PC. As shown in Fig 3A, the synthesis of PC *in vivo* via this pathway was reduced to 60% of wild type. Interestingly, however, also the methylation pathway was affected by the Δ*gph1* deletion (Fig 3B). In this assay, cells were labeled with L-[^3^H]serine, and sequential incorporation of the label into PS, PE (catalyzed by Psd1p or Psd2p), and PC (catalyzed by Cho2p and Opi3p) was measured. Whereas the first two steps in the biosynthetic route of aminoglycerophospholipids were reduced by 20%, the methylation of PE to PC in Δ*gph1* was approximately only 50% of wild type. In summary, formation of PC through both pathways was strongly decreased in the mutant.

As diacylglycerol and glycerol 3-phosphate are precursors of PC their intracellular concentration was considered to have a possible influence on the cellular PC level [2, 4]. However, down regulation of PC formation in the *Δgph1* deletion strain could not be attributed to the limited availability of diacylglycerol or glycerol-3-phosphate. We found that the glycerol-3-phosphate content of the *Δgph1* mutant strain (2.412 ± 0.510 nmol/μl cell lysate) was even more the ten times higher than in wild type (0.196 ± 0.045 nmol/μl cell lysate), and also the DG content in the *Δgph1* strain (0.068 ± 0.016 μg/OD_600_ unit) was higher than in wild type (0.053 ± 0.002 μg/OD_600_ unit). These results showed that the two components were not limiting for the synthesis of PC in the *Δgph1* mutant in *vivo*.

**Fig 4:**
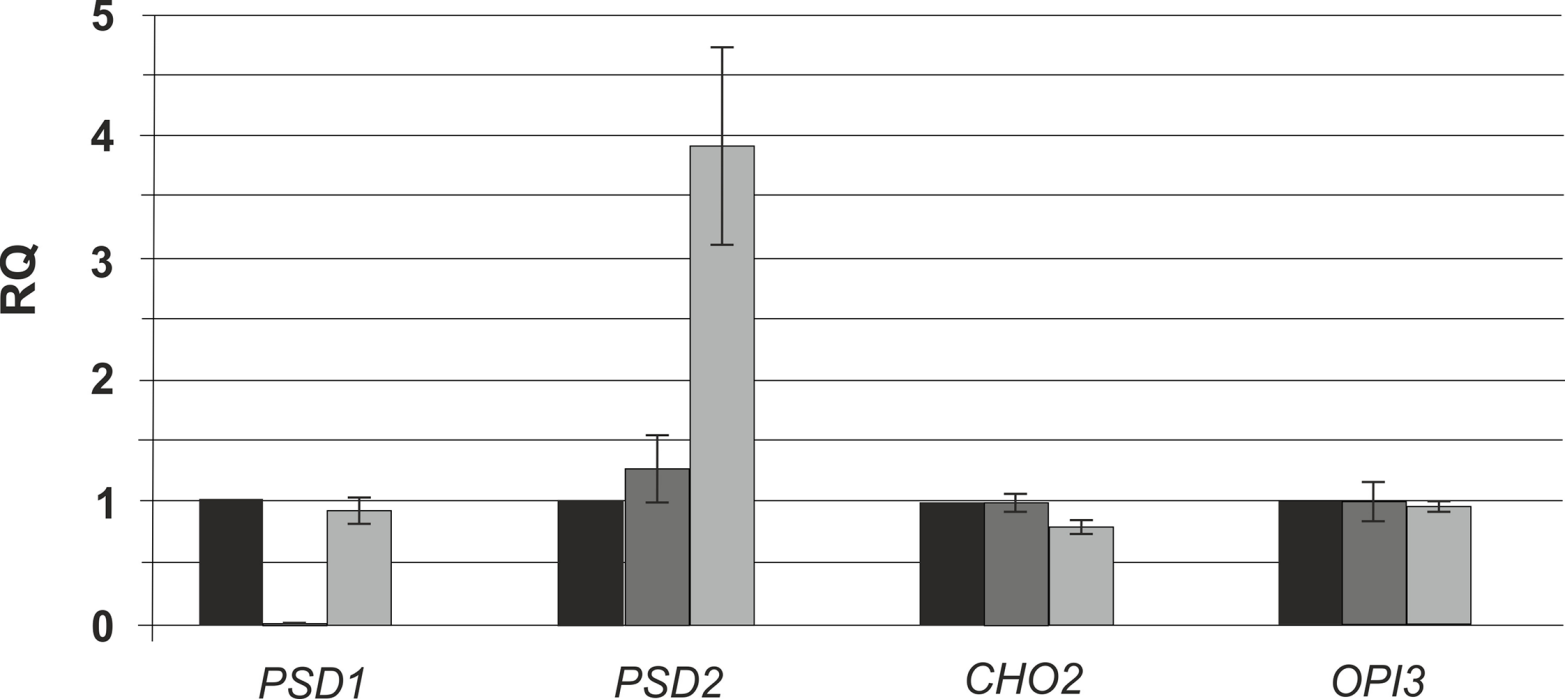
Expression levels of *PSD1*, *PSD2*, *CHO2* and *OPI3* in the Δ*gph1* deletion mutant. Relative gene expression of *PSD1*, *PSD2*, *CHO2* and *OPI3* in the Δ*gph1* deletion mutant was measured by RT-qPCR. A *Δpsd1* mutant was used as a negative control. Wild type was set at 1. Data are mean values from three independent experiments with the respective deviation.

We also tested the transcription rate of genes encoding key enzymes of aminoglycerophospholipid metabolism in wild type and Δ*gph1*. A Δ*psd1* deletion mutant was used as a negative control. As can be seen from Fig 4, genes encoding the phospholipid methyltransferase Cho2p and Opi3p as well as the PS decarboxylase Psd1p were not affected by the Δ*gph1* mutation. Interestingly, transcription of *PSD2* was strongly increased in the Δ*gph1* mutant. We may interpret this effect as compensation for the depleted activities of the aminoglycerophospholipid biosynthetic pathway (see Fig 3). Such compensation, however, was not observed with a Δ*psd1* mutant strain.

**Fig 5:**
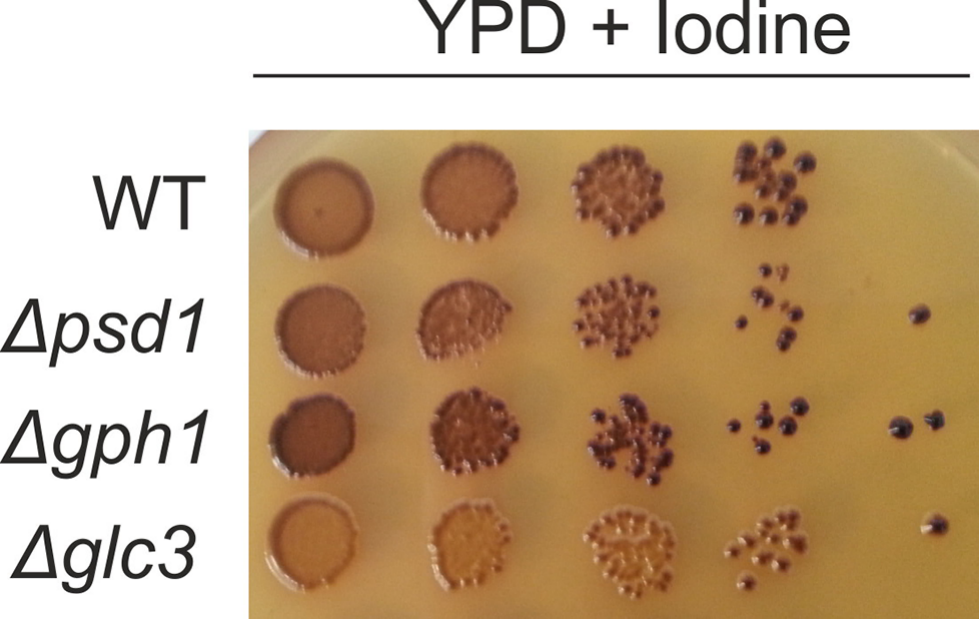
Glycogen content in the *Δgph1*, *Δpsd1* and *Δglc3* deletion mutants. Strains as indicated were grown on YPD plates. Cell suspensions were spotted at dilutions 1, 1/10, 1/100, 1/1000, 1/10000 and incubated at 30°C for 48 h. Plates were exposed to iodine vapor for 10 min.

Finally, we tested whether inhibition of PC synthesis (see Fig 3) was linked to the accumulation of glycogen in the *Δgph1* deletion mutant. Fig 5 shows the glycogen content of *Δgph1*, *Δpsd1* and *Δglc3* deletion mutants compared to the wild type *BY4741*. As expected, in the *Δgph1* strain glycogen accumulated, whereas in the *Δglc3* strain glycogen synthesis was inhibited. As *Δgph1* leads to a decrease of PC [14], and *Δglc3* to an increased PC level, one might argue that high glycogen levels correlate with low PC levels and vice versa. However, the fact that *sga1Δ* and *gdb1Δ* deletions, which also lead to the accumulation of glycogen, do not result in a significant change of PC (see above), supports the conclusion that there is no general correlation between glycogen and PC levels.

### Metabolism of non-polar lipids in the *Δgph1* mutant

**Fig 6:**
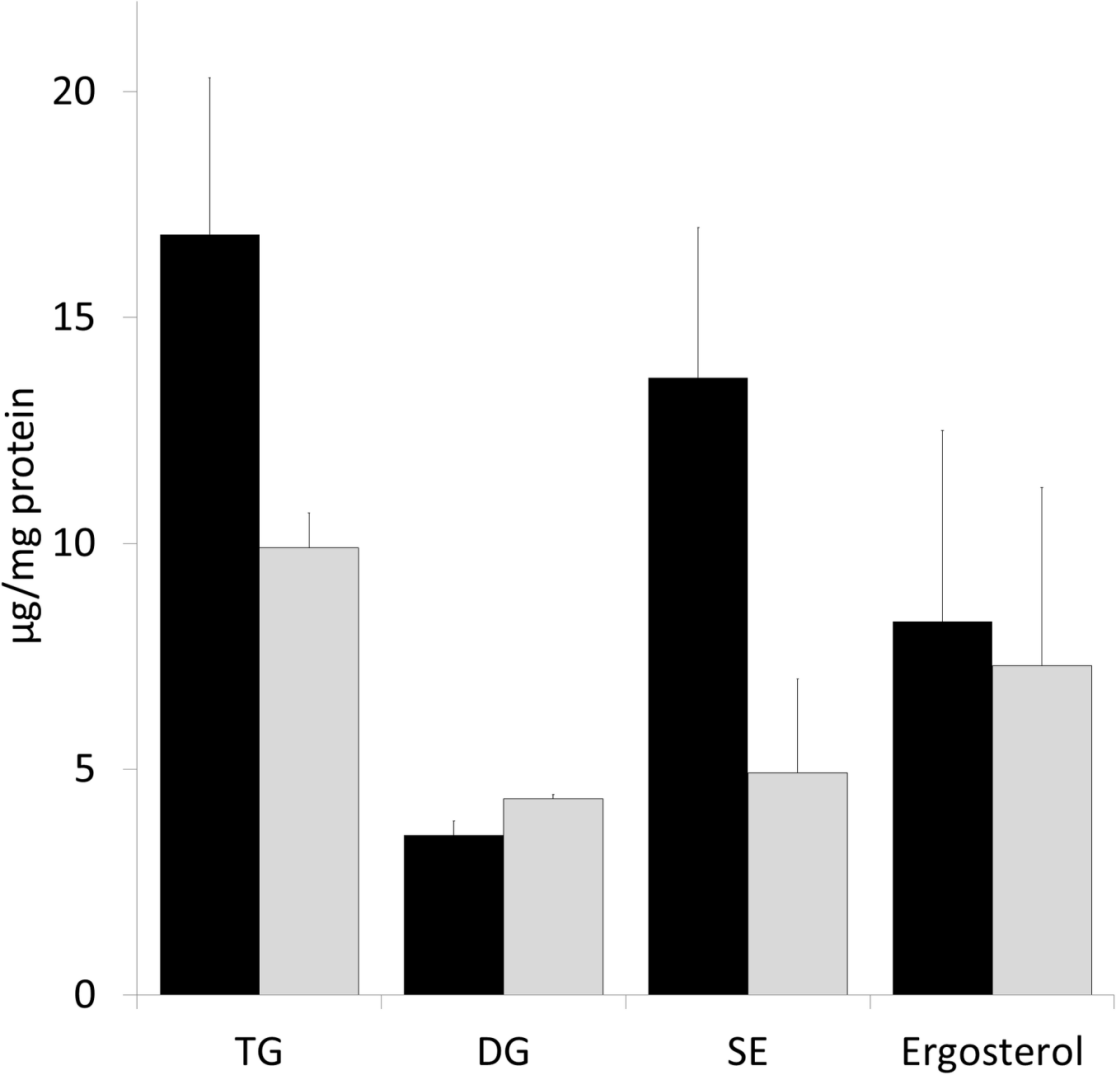
Amounts of triacylglycerols and steryl esters are reduced in the Δ*gph1* deletion mutant. The wild type BY4741 (black bar) and the Δ*gph1* deletion mutant (grey bar) were grown aerobically to the early stationary growth phase, and triacylglycerols (TG), diacylglycerols (DG), steryl esters (SE) and ergosterol were quantified. Results were obtained from 3 independent samples with deviations as indicated by the error bars.

Changes in the phospholipid profile tempted us to speculate that also non-polar lipid metabolism was affected in the Δ*gph1* deletion strain. As can be seen from Fig 6 the amounts of TG and SE were indeed markedly reduced in the Δ*gph1* mutant. At the same time, the level of DG in the Δ*gph1* mutant was slightly increased (see also above) over wild type suggesting a possible involvement of Gph1p in the synthesis of TG. The amount of ergosterol was more or less the same in the mutant and in wild type.

**Fig 7:**
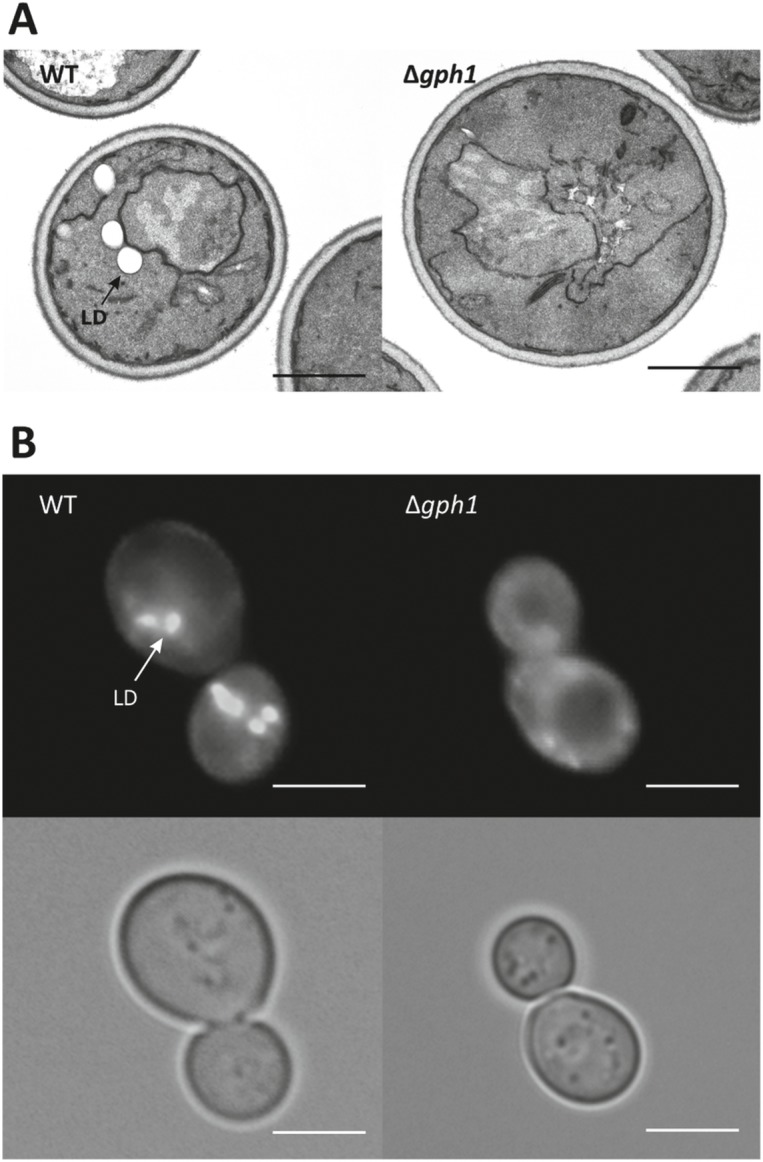
Number and size of lipid droplets are decreased in the Δ*gph1* deletion mutant. (A) Transmission electron microscopy images of wild type BY4741 and Δ*gph1*. (B) Nile Red staining and fluorescence microscopy of wild type BY4741 and Δ*gph1*. LD: lipid droplets. Scale bar: 2 μm.

As TG and SE are the main components of lipid droplets we also investigated the effect of the Δ*gph1* deletion on the formation of this compartment. Electron microscopic analysis of the Δ*gph1* mutant showed to our surprise that lipid droplets were more or less missing in the Δ*gph1* mutant strain (Fig 7A). In the wild type strain one to four distinct lipid droplets were detected per cell, whereas the Δ*gph1* deletion mutant lacked lipid droplets or contained droplets with very small size. This observation was confirmed by Nile Red staining and fluorescence microscopy (Fig 7B). The typical fluorescence signals of lipid droplets stained with Nile Red appearing as distinct spots were only detected in wild type, whereas in Δ*gph1* only diffuse fluorescence signals in the cellular background were observed.

Electron microscopic inspection of the Δ*gph1* mutant (see Fig 7A) also revealed that the total cell structure including the plasma membrane was not changed. High sensitivity of the deletion mutant to SDS [14] had been a hint for possible structural defects of the plasma membrane. However, such changes were not observed.

**Fig 8:**
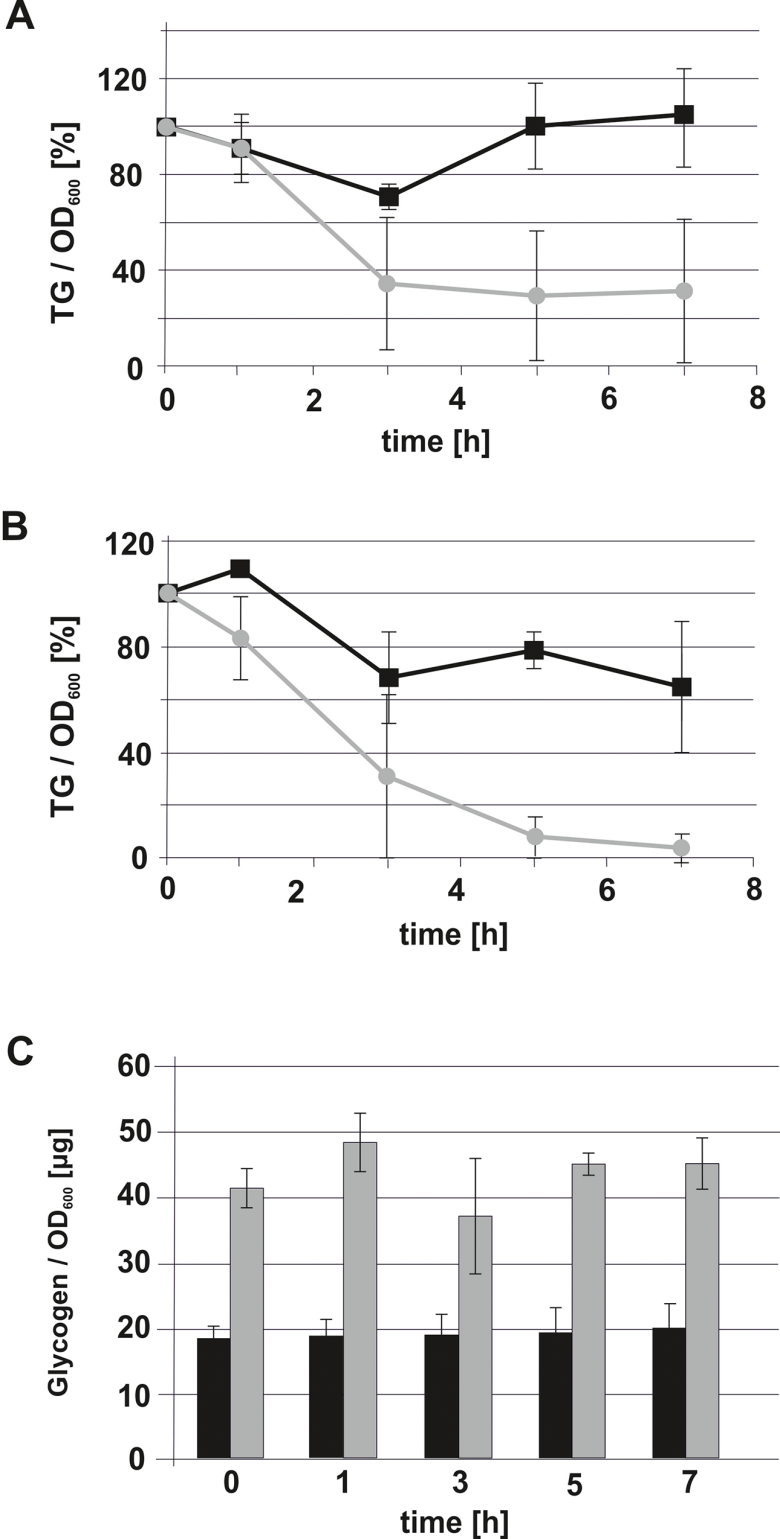
Triacylglycerol degradation in the *Δgph1* mutant. Wild type BY4741 (A) and *Δgph1* mutant cells (B) were grown to an OD_600_ of 3. Cerulenin was added at time point 0 to a final concentration of 10 μg/μl (grey line; -●-). The black line (-■-) shows the control without cerulenin. Over the time period of TG degradation the amount of glycogen was tested in wild type BY4741 (black bars) and *Δgph1* (grey bars). Amounts of glycogen were expressed as μg/OD_600_ unit. Results were obtained from 3 independent samples with deviations as indicated by the error bars.

The question remained whether an increased turnover of TG in the Δ*gph1* mutant was the reason for the decreased amount of this lipid in the mutant. To address this question, we performed mobilization assays of cellular TG *in vivo* in the presence of cerulenin, an inhibitor of fatty acid synthesis in yeast [40]. Under these conditions, fatty acids from TG get mobilized mainly to be incorporated into membrane phospholipids [45]. In both wild type and Δ*gph1* TG was properly mobilized, although the initial TG degradation rate in the mutant was slightly higher (Fig 8A and 8B). Thus, changed TG hydrolysis in Δ*gph1* did not contribute to the lower level of this lipid in the mutant. To correlate the glycogen content with TG degradation we also measured the concentration of glycogen over the time period of TG mobilization. As can be seen from Fig 8C, the glycogen levels in the two tested strains were more or less constant during TG degradation. However, the absolute amounts of glycogen in wild type and *Δgph1* were different as expected. In wild type, approximately 20 μg glycogen per OD_600_ unit were detected, whereas in the mutant the glycogen level was approximately 45 μg per OD_600_ unit over the inspected time period. This result suggested that TG degradation and glycogen storage occurred independently of each other, at least under cultivation conditions described here.

**Fig 9:**
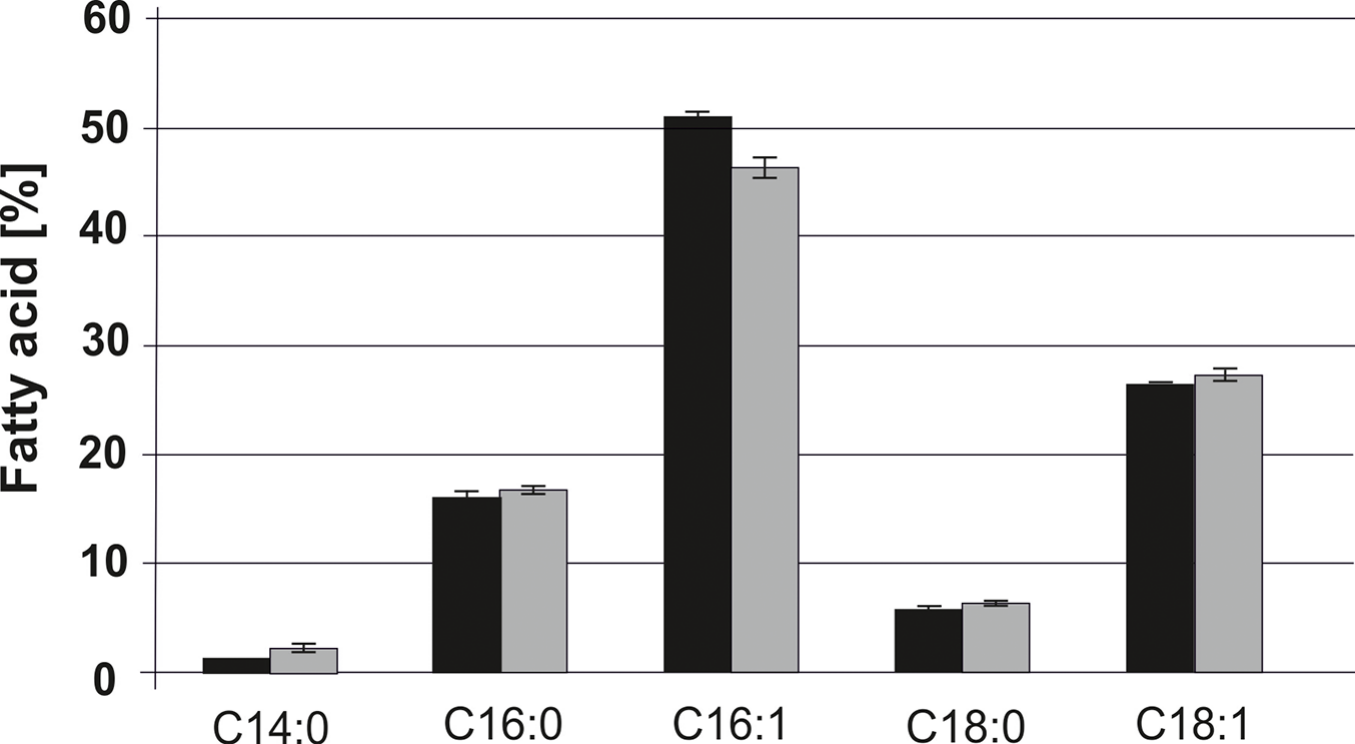
Fatty acid profile of the *Δgph1* mutant. The distribution of myristic acid (C14:0), palmitic acid (C16:0), palmitoleic acid (C16:1), stearic acid (C18:0) and oleic acid (C18:1) was analyzed in wild type BY4741 (black bar) and in the *Δgph1* strain (grey bar). Results were obtained from 3 independent samples with deviations as indicated by the error bars.

Changes in the patterns of phospholipids and non-polar lipids from the Δ*gph1* mutant further led us to examine a possible role of Gph1p in fatty acid synthesis. To address this question we analyzed the fatty acid profile from wild type BY4741 and *Δgph1*. As can be seen from Fig 9 the *Δgph1* mutation did not affect the cellular fatty acids pattern. Thus, this branch of lipid metabolism was obviously not influenced by GPH1.

## Discussion

Lipid metabolism of the yeast is a complex network of reactions with an even more complicated regulatory background. Besides genes encoding lipid metabolic enzymes a number of regulatory genes whose products are involved in synthesis and metabolic conversion of lipids have been identified (reviewed in ref. [4]). Consequently, synthesis and metabolism of the major yeast lipid classes, e.g. phospholipids, fatty acids, TG, sterols and sphingolipids are linked to each other [2]. Additionally, lipid synthesis in yeast is affected by growth conditions which influence the expression of enzymes and/or modulate their catalytic activities. As examples, expression of phospholipid biosynthetic genes in yeast is affected by carbon sources, availability of nutrient, growth phase, pH and temperature. Finally, posttranslational modifications of gene products, especially phosphorylation of key proteins involved in phospholipid synthesis, may affect metabolism of phospholipids and the balance between certain lipid precursors and final products of lipid biosynthetic pathways [5,46–51].

In previous studies from our laboratory [14] *GPH1* was identified as possible regulator gene of yeast lipid metabolism. Gph1p has originally been identified as a glycogen phosphorylase which catabolizes the branched polysaccharide glycogen used as storage carbohydrate [15–17]. In this study, we present extended evidence about the influence of *GPH1* expression on lipid metabolism in yeast. Besides the previously described effect of a Δ*gph1* deletion on PC synthesis [14] we show here that a mutant deleted of *GPH1* exhibited decreased formation of TG and SE, but increased synthesis of total phospholipids (see Figs 2, 3 and 6). Depletion of TG and SE in Δ*gph1* mutant cells led to lack of lipid droplets (see Fig 7). Changes in the phospholipid composition, especially in the plasma membrane, were most likely the reason for the increased sensitivity of *Δgph1* against SDS. This view was confirmed by our findings that a *Δcho2Δopi3/Δpem1Δpem2* mutant which was strongly depleted of PC, also showed SDS sensitivity (S1 Fig). Slight sensitivity of the *Δcho2Δopi3* mutant against Calcofluor White was also observed. Thus, PC depletion obviously leads to defects in the yeast cell periphery.

Based on the results described above we speculated about a link between carbohydrate metabolism of the yeast (glycogen storage and mobilization) and lipid metabolism through the action of Gph1p. For this reason, we first analyzed a *Δglc3* mutant strain. Glc3p is also an enzyme of glycogen metabolism, although in the biosynthetic branch. The *Δglc3* mutation caused only minor changes in the pattern of total cellular lipids (see Table 3). In mitochondria from *Δglc3*, a marked shift to lower PC and higher PE values was observed, whereas in the plasma membrane the opposite effect was seen. Furthermore, we also tested possible effects of *sga1Δ* and *gdb1Δ* deletions on lipid metabolism. Both the *SGA1* and the *GDB1* gene products are involved in glycogen metabolism similar to Gph1p. However, lipid profiles of *Δsga1* and *Δgdb1* deletion strains were similar to wild type indicating that the effect of Δ*gph1* was specific and unique. Altogether, we did not find a clear correlation between glycogen and PC metabolism in the yeast. Thus, Gph1p may fulfill multiple independent functions which affect carbohydrate metabolism on the one hand and lipid metabolism on the other hand.

Our investigations also addressed a possible role of Gph1p as a regulator of yeast TG lipases (see Fig 8). The decreased TG in the Δ*gph1* deletion mutant was regarded as a possible result of such an effect. However, only minor differences in TG degradation were found in wild type and in the Δ*gph1* deletion mutant. Moreover, expression levels of the three yeast genes encoding TG lipases, *TGL3*, *TGL4* and *TGL5*, were not changed in the Δ*gph1* strain (data not shown). Depletion of TG and SE are obviously the reason for the surprising observation that the Δ*gph1* mutant lacks lipid droplets. It is even more surprising that lipid droplets are not formed although some TG and SE are still present in the mutant cells (see Figs 6 and 7). It can only be speculated that the remaining amounts of TG and SE are spread over internal membranes without leading to initiation of lipid droplet formation.

The molecular role of Gph1p in lipid metabolism is subject to speculation. Gph1p is a phosphatase and degrades glycogen leading to the formation of glucose-1-phosphate. Evidence presented here suggests, however, that Gph1p has more than one function in cell metabolism. Examples for such enzymes in lipid metabolism are the yeast TG lipases Tgl3p, Tgl4p and Tgl5p which serve simultaneously as lipases and acyltransferases [52]. It has been shown before that Gph1p is mostly active in the stationary phase of a yeast culture and not even properly expressed earlier [15]. In contrast, our study demonstrated a number of effects occurring already in earlier growth phases, e.g., sensitivity to SDS, changes in the lipid pattern, and lipid formation *in vivo*. These results largely exclude that glycogen accumulation directly affects the lipid metabolism, because glycogen accumulation does not occur in early growth phases.

Finally, we may speculate that Gph1p affects the subcellular distribution of lipid components. One piece of evidence in this line is the fact that TG, although formed at reduced quantities in Δ*gph1*, is not stored in lipid droplets at substantial amounts. The other finding supporting this view is that deletion of *GPH1* strongly and at least to some extend specifically affects assembly of PC into the plasma membrane. Altogether, Gph1p appears to cause several changes in yeast lipid metabolism whose molecular mechanisms remain to be elucidated.

## Supporting Information

S1 Fig Sodium dodecyl sulfate and Calcofluor White sensitivity of *Δcho2Δopi3*, *Δcki1Δdpl1Δeki3* and *Δgph1* deletion mutants. Wild type BY4741 and mutant strains as indicated were grown on YPD plates and on YPD plates containing 0.05% sodium dodecyl sulfate (SDS) or 20 μg/ml Calcofluor White (CFW), respectively. The double mutant *Δcho2Δopi3* is blocked in the methylation pathway of PC synthesis, and the *Δcki1Δdpl1Δeki3* triple mutant is blocked in the CDP-choline pathway of PC synthesis.
(TIF)
