# Supplementary figures and images for: A Yeast Mutant Deleted of GPH1 Bears Defects in Lipid Metabolism
Source: PLoS One. 2015 Sep 1;10(9):e0136957. doi: 10.1371/journal.pone.0136957 (PMC4556709; doi:10.1371/journal.pone.0136957)

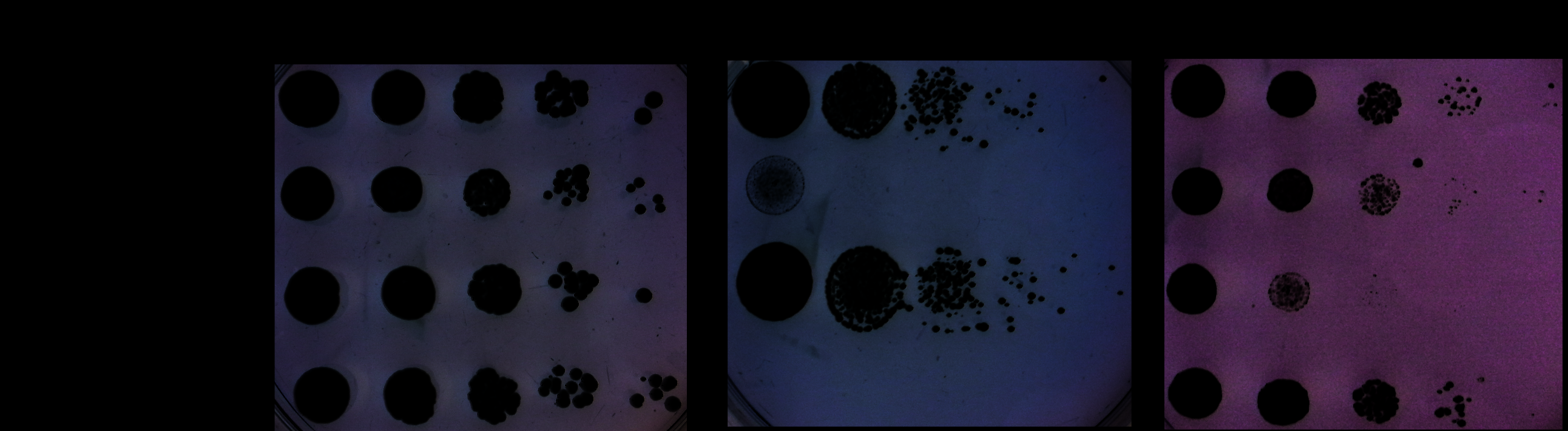

Supplement: S1 Fig — Wild type BY4741 and mutant strains as indicated were grown on YPD plates and on YPD plates containing 0.05% sodium dodecyl sulfate (SDS) or 20 μg/ml Calcofluor White (CFW), respectively. The double mutant Δcho2Δopi3 is blocked in the methylation pathway of PC synthesis, and the Δcki1Δdpl1Δeki3 triple mutant is blocked in the CDP-choline pathway of PC synthesis. (TIF) [file pone.0136957.s001.tif]
